# Supplementary material for: Quantifying the impacts of volume-based procurement policy on spatial accessibility of antidepressants via generic substitution: A four-city cohort study using drug sales data
Source: PLoS One. 2025 Feb 10;20(2):e0318509. doi: 10.1371/journal.pone.0318509 (PMC11809876; doi:10.1371/journal.pone.0318509)
Supplement: S1 Table — The public drug information in the table is sourced from https://www.smpaa.cn/. (DOCX) [file pone.0318509.s001.docx]

**S1 Table:** Characteristics of Antidepressant Medications Included in the VBP.

| **Antidepressants** | **Type** | **VBP batch** | **VBP time** | **Common dosage forms** |
| --- | --- | --- | --- | --- |
| Escitalopram | SSRIs | 1st | 2019/9 | Oral regular release dosage form |
| Paroxetine | SSRIs | 1st | 2019/9 | Tablet |
| Fluoxetine | SSRIs | 3rd | 2020/8 | Capsules |
| Citalopram | SSRIs | 3rd | 2020/8 | Oral regular release dosage form |
| Sertraline | SSRIs | 3rd | 2020/8 | Oral regular release dosage form |
| Duloxetine | SNRIs | 4th | 2021/2 | Oral regular release dosage form |
| Venlafaxine | SNRIs | 5th | 2021/6 | Oral sustained-release and controlled-release form |
| Flupentixol-melitracen | TcAs | 7th | 2022/7 | Oral regular release dosage form |
| Mirtazapine | NaSSAs | 8th | 2023/4 | Oral regular release dosage form |

^a^ The public drug information in the table is sourced from <https://www.smpaa.cn/>.
