# Supplementary material for: Quantifying the impacts of volume-based procurement policy on spatial accessibility of antidepressants via generic substitution: A four-city cohort study using drug sales data
Source: PLoS One. 2025 Feb 10;20(2):e0318509. doi: 10.1371/journal.pone.0318509 (PMC11809876; doi:10.1371/journal.pone.0318509)
Supplement: S2 Table — The administrative boundary is sourced from the Chinese Administration of Surveying Mapping and Geoinformation, with review number GS (2022) 399. (DOCX) [file pone.0318509.s002.docx]

**S2 Table:** Datasets Used in this National Study in China.

| **Dataset** | **Year** | **Source** |
| --- | --- | --- |
| Drug procurement information | 2018-2020 | China Pharmaceutical Association |
| Population information | 2019-2021 | the National Statistical Yearbook of 2019-2021 (https://www.stats.gov.cn) |
| Include hospital, Road network, Point of interest (POI) | 2018-2020 | Gaode Location Service and Road Traffic Network (https://www.gaode.com) |
| Administrative boundary | 2022 | Chinese Administration of Surveying Mapping and Geoinformation |
| Population count data | 2020 | WorldPop (https://hub.worldpop.org) |
| Friction surfaces for calculating travel time | 2020 | the Malaria Atlas Project (https://malariaatlas.org) |

^a^ The administrative boundary is sourced from the Chinese Administration of Surveying Mapping and Geoinformation, with review number GS (2022) 399.
