# Supplementary material for: Quantifying the impacts of volume-based procurement policy on spatial accessibility of antidepressants via generic substitution: A four-city cohort study using drug sales data
Source: PLoS One. 2025 Feb 10;20(2):e0318509. doi: 10.1371/journal.pone.0318509 (PMC11809876; doi:10.1371/journal.pone.0318509)
Supplement: S3 Table — The proportion of generic drugs is calculated based on procurement quantity. (DOCX) [file pone.0318509.s003.docx]

**S3 Table:** Proportion of Generic Drugs by Quarter (%).

|  | **2018Q1** | **2018Q2** | **2018Q3** | **2018Q4** | **2019Q1** | **2019Q2** | **2019Q3** | **2019Q4** | **2020Q1** | **2020Q2** | **2020Q3** | **2020Q4** |
| --- | --- | --- | --- | --- | --- | --- | --- | --- | --- | --- | --- | --- |
| **Escitalopram** | | | | | | | | | | | | |
| Beijing | 0.45429 | 0.50032 | 0.50209 | 0.53694 | 0.55202 | 0.62438 | 0.61979 | 0.67107 | 0.62465 | 0.65762 | 0.68166 | 0.63138 |
| Shanghai | 0.60594 | 0.57685 | 0.56853 | 0.52688 | 0.63478 | 0.73579 | 0.75568 | 0.80234 | 0.80228 | 0.80780 | 0.78130 | 0.76235 |
| Ningbo | 0.65019 | 0.67325 | 0.67239 | 0.68937 | 0.71134 | 0.71587 | 0.73262 | 0.77895 | 0.83379 | 0.86146 | 0.85191 | 0.82189 |
| Harbin | 0.39372 | 0.31534 | 0.26446 | 0.29975 | 0.43966 | 0.44013 | 0.31236 | 0.30453 | 0.40145 | 0.46891 | 0.33555 | 0.88898 |
| **Paroxetine** | | | | | | | | | | | | |
| Beijing | 0.60688 | 0.62722 | 0.65816 | 0.60991 | 0.69525 | 0.68231 | 0.73179 | 0.74673 | 0.78639 | 0.84608 | 0.82214 | 0.81713 |
| Shanghai | 0.81823 | 0.82495 | 0.81465 | 0.81097 | 0.84863 | 0.86229 | 0.86731 | 0.87105 | 0.84641 | 0.84028 | 0.84897 | 0.85380 |
| Ningbo | 0.80328 | 0.82713 | 0.82161 | 0.83013 | 0.78736 | 0.78186 | 0.79120 | 0.88282 | 0.87750 | 0.88339 | 0.84166 | 0.84078 |
| Harbin | 0.00000 | 0.00000 | 0.00000 | 0.09638 | 0.14248 | 0.09481 | 0.19366 | 0.61759 | 0.83860 | 0.72896 | 0.77248 | 0.89415 |

^a^ The proportion of generic drugs is calculated based on procurement quantity.
