# Supplementary material for: Quantifying the impacts of volume-based procurement policy on spatial accessibility of antidepressants via generic substitution: A four-city cohort study using drug sales data
Source: PLoS One. 2025 Feb 10;20(2):e0318509. doi: 10.1371/journal.pone.0318509 (PMC11809876; doi:10.1371/journal.pone.0318509)
Supplement: S4 Table — ACME: The average causal mediation effects; ADE: Average direct effects. (DOCX) [file pone.0318509.s004.docx]

**S4 Table:** Adjusted Regression Coefficients and Mediation Analysis of Generic Drug Proportion in the Spatial Inequality of Antidepressants.

|  | **Average Gini index before policy** | **Average Gini index after policy** | **Average Generic percentage before policy** | **Average Generic percentage after policy** | **Policy impacts on Gini index**  **(Beta)** | **P-value** | **Policy impacts on generic percentage (Beta)** | **P-value** | **Generic percentage on Gini index**  **(Beta)** | **P-value** | **Proportion mediated by generic percentage, %（95%CI）** | **ACME P-value** | **ADE P-value** |
| --- | --- | --- | --- | --- | --- | --- | --- | --- | --- | --- | --- | --- | --- |
| **Escitalopram** | | | | | | | | | | | | | |
| Beijing | 0.013 | 0.010 | 0.504 | 0.643 | -0.003 | 0.000 | 0.138 | 0.000 | -0.019 | 0.000 | 0.480（0.078~0.940） | -0.001  （0.010） | -0.001  （0.024） |
| Shanghai | 0.010 | 0.010 | 0.585 | 0.778 | -0.000 | 0.971 | 0.193 | 0.000 | -0.001 | 0.781 | 0.095（-3.45~3.65） | -0.000  （0.780） | -0.000  （0.280） |
| Ningbo | 0.031 | 0.028 | 0.707 | 0.842 | -0.003 | 0.010 | 0.135 | 0.000 | -0.014 | 0.043 | -0.002（-1.61~1.45） | -0.000  （0.994） | -0.003  （0.106） |
| Harbin | 0.378 | 0.386 | 0.357 | 0.516 | 0.007 | 0.298 | 0.158 | 0.009 | 0.013 | 0.507 | 0.052（-4.711~3.77） | 0.000  （0.830） | 0.006  （0.420） |
| **Paroxetine** | | | | | | | | | | | | | |
| Beijing | 0.012 | 0.011 | 0.632 | 0.772 | -0.001 | 0.005 | 0.140 | 0.000 | -0.009 | 0.001 | 0.644（-0.186~2.270） | -0.000  （0.124） | -0.000  （0.506） |
| Shanghai | 0.006 | 0.006 | 0.822 | 0.855 | -0.000 | 0.137 | 0.033 | 0.000 | -0.007 | 0.261 | 0.121（-2.53~3.98） | -0.000  （0.750） | -0.000  （0.290） |
| Ningbo | 0.030 | 0.028 | 0.815 | 0.854 | -0.001 | 0.026 | 0.039 | 0.013 | -0.003 | 0.670 | -0.074（-0.824~0.45） | 0.000  （0.588） | -0.002  （0.014） |
| Harbin | 0.395 | 0.366 | 0.095 | 0.813 | -0.029 | 0.000 | 0.718 | 0.000 | -0.045 | 0.000 | 1.536（0.851~3.14） | -0.045  （0.000） | 0.016  （0.140） |

^a^ ACME: The average causal mediation effects; ADE: Average direct effects.
