# Supplementary figures and images for: Quantifying the impacts of volume-based procurement policy on spatial accessibility of antidepressants via generic substitution: A four-city cohort study using drug sales data
Source: PLoS One. 2025 Feb 10;20(2):e0318509. doi: 10.1371/journal.pone.0318509 (PMC11809876; doi:10.1371/journal.pone.0318509)

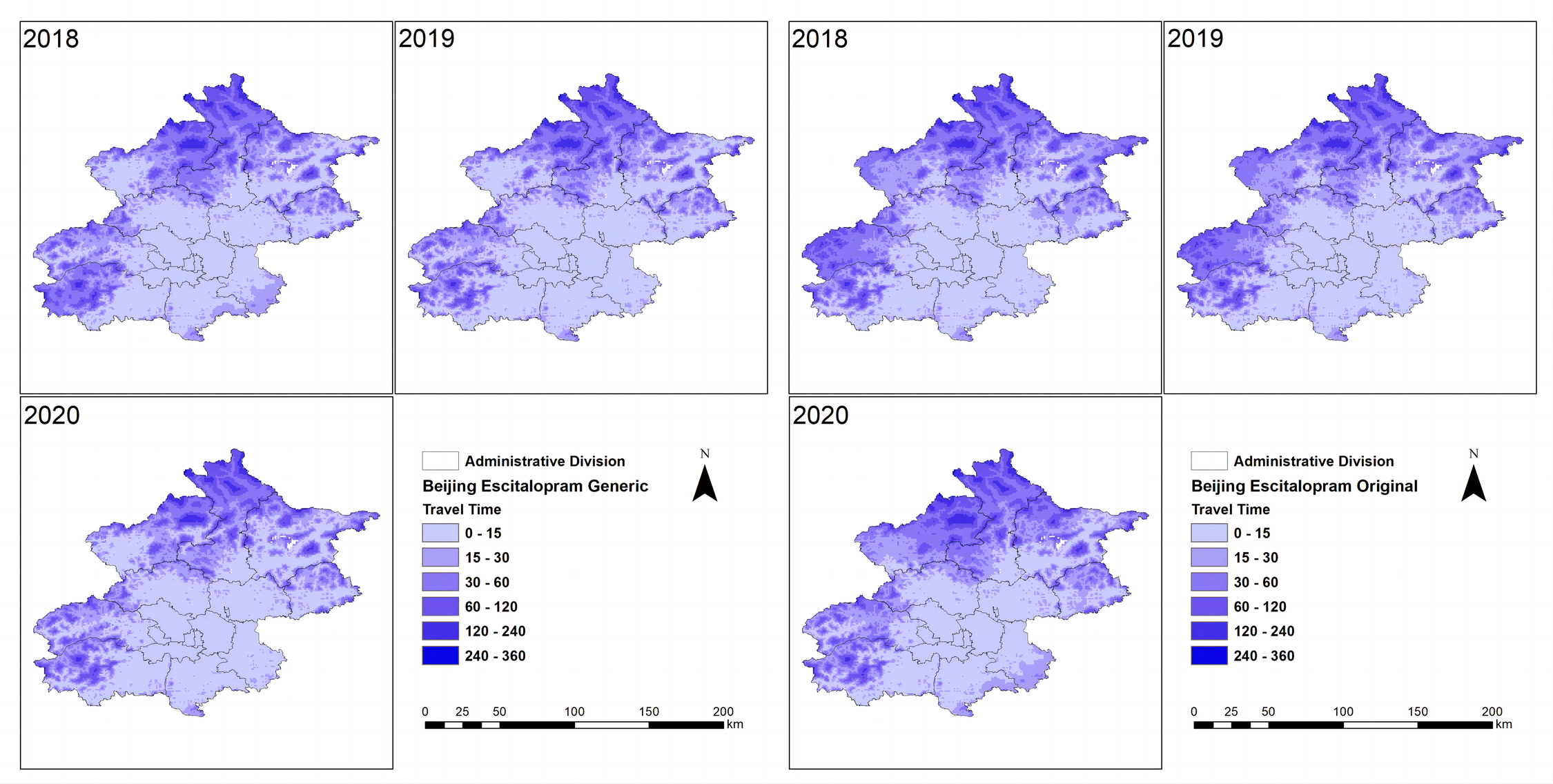

Supplement: S1 Fig — The shortest travel time from each 1 km2 population point to the nearest mental healthcare of Escitalopram in Beijing was categorized into the following intervals: 0–15, 15–30, 30–60, 60–120, 120–240, and 240–360 minutes. (TIF) [file pone.0318509.s005.tif]

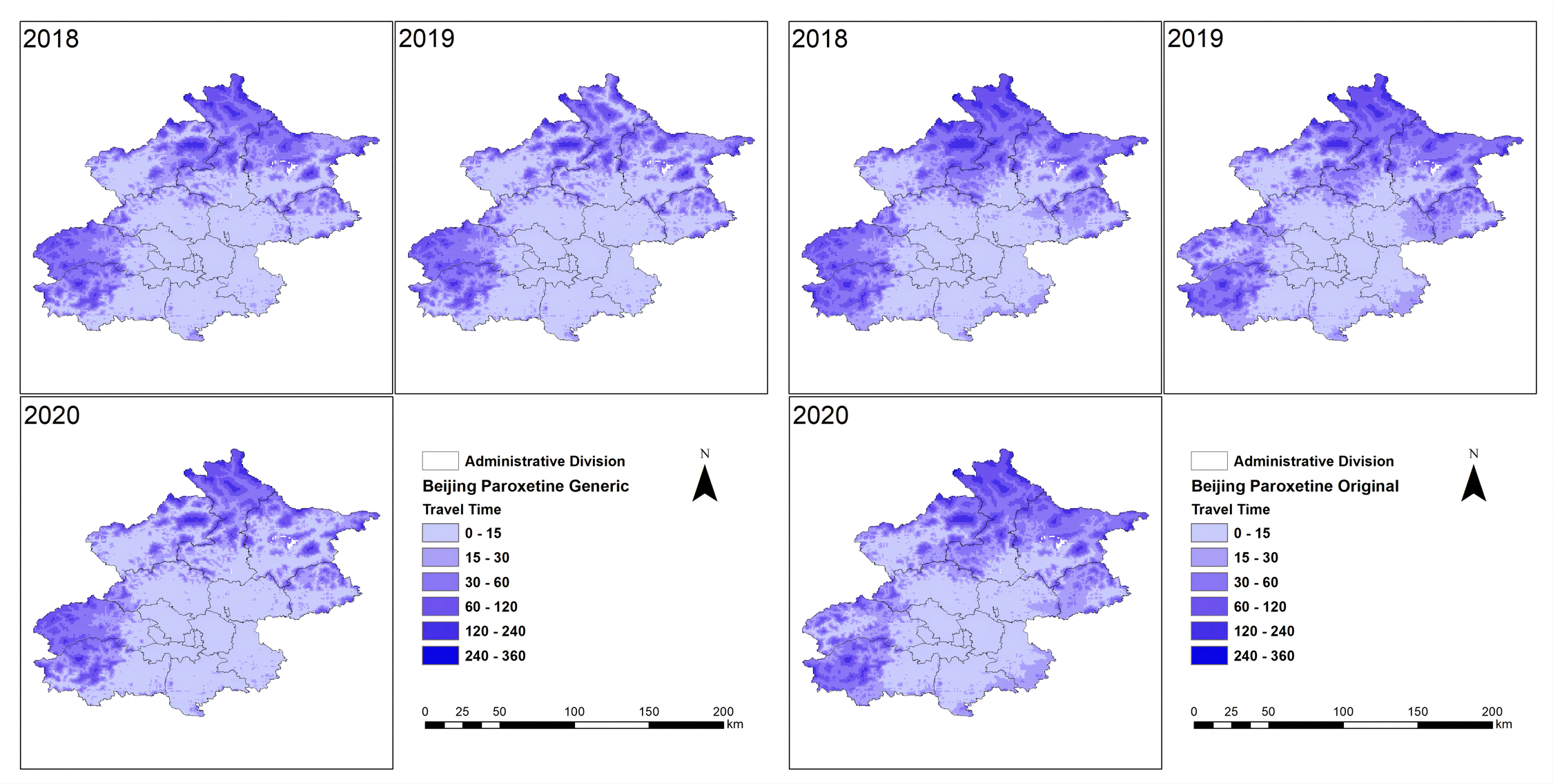

Supplement: S2 Fig — The shortest travel time from each 1 km2 population point to the nearest mental healthcare of Paroxetine in Beijing was categorized into the following intervals: 0–15, 15–30, 30–60, 60–120, 120–240, and 240–360 minutes. (TIF) [file pone.0318509.s006.tif]

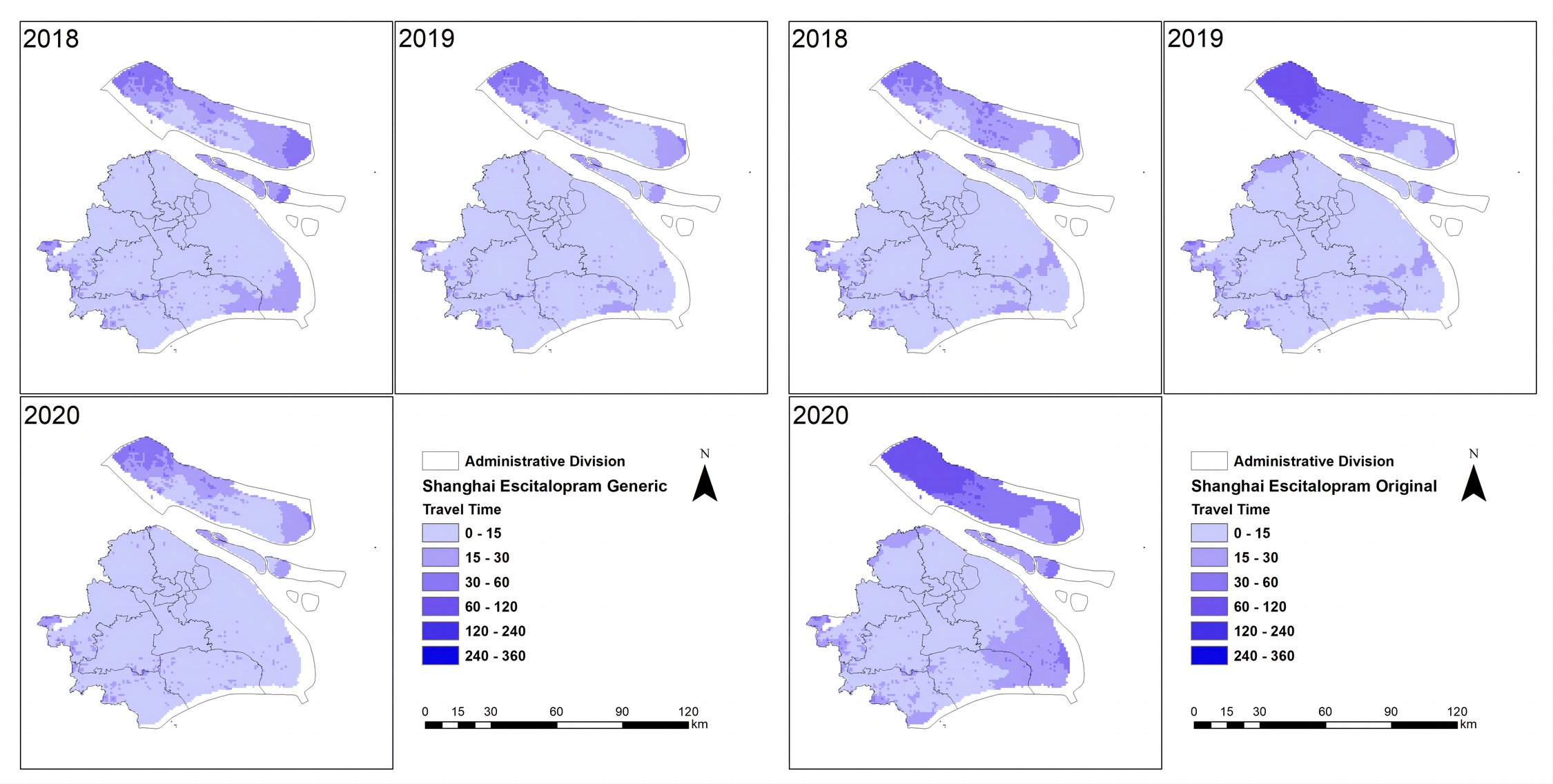

Supplement: S3 Fig — The shortest travel time from each 1 km2 population point to the nearest mental healthcare of Escitalopram in Shanghai was categorized into the following intervals: 0–15, 15–30, 30–60, 60–120, 120–240, and 240–360 minutes. (TIF) [file pone.0318509.s007.tif]

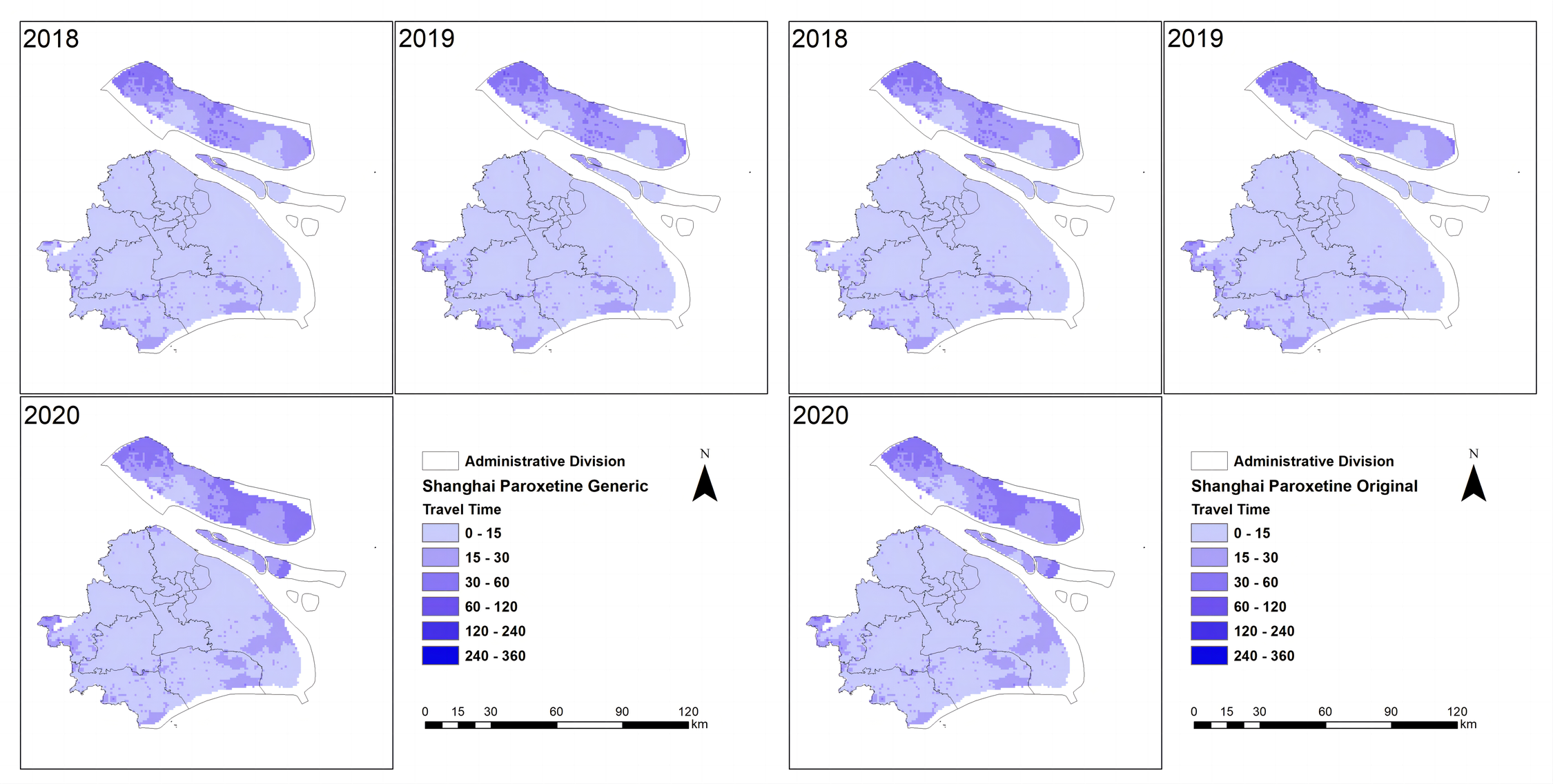

Supplement: S4 Fig — The shortest travel time from each 1 km2 population point to the nearest mental healthcare of Paroxetine in Shanghai was categorized into the following intervals: 0–15, 15–30, 30–60, 60–120, 120–240, and 240–360 minutes. (TIF) [file pone.0318509.s008.tif]

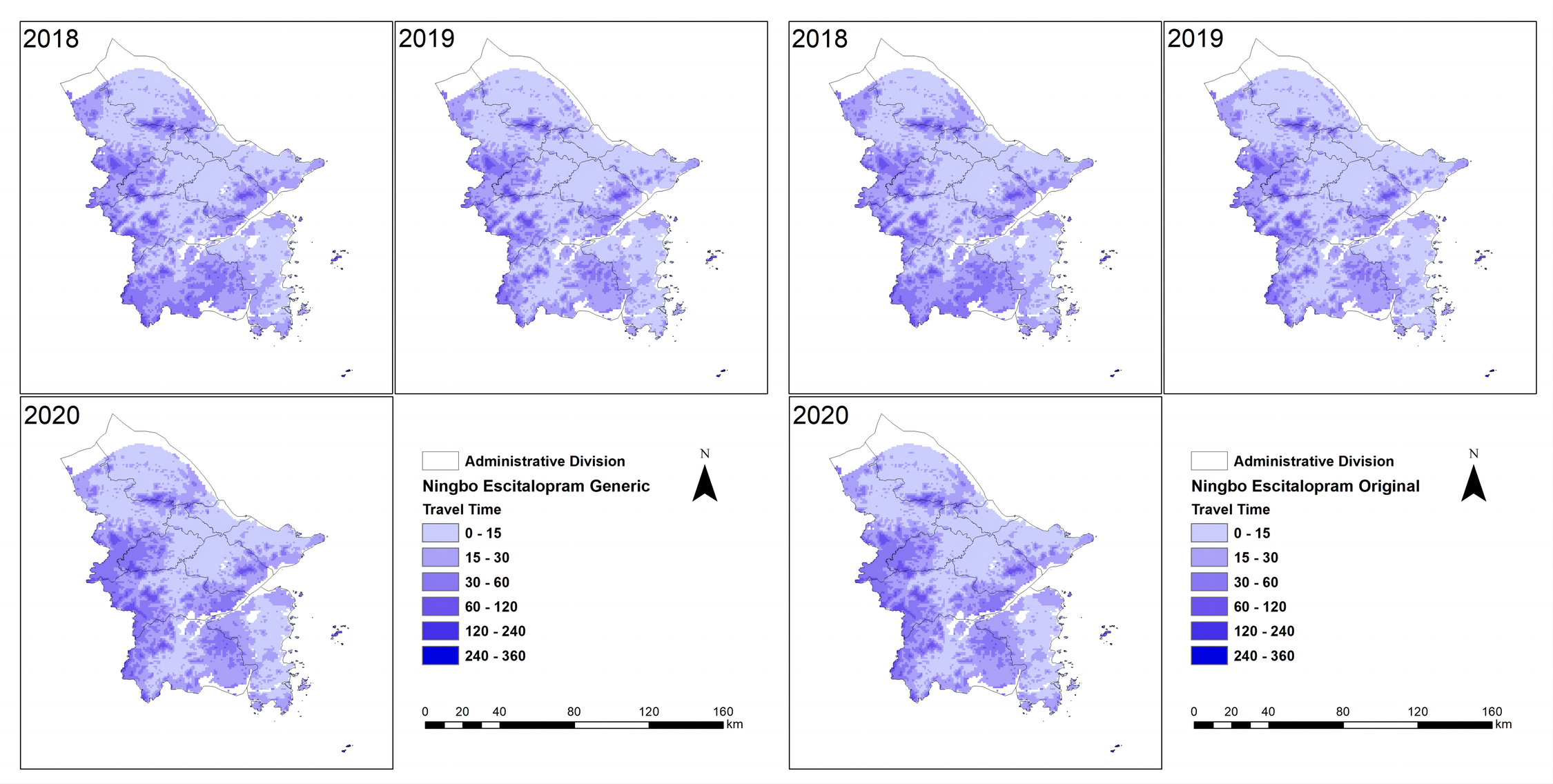

Supplement: S5 Fig — The shortest travel time from each 1 km2 population point to the nearest mental healthcare of Escitalopram in Ningbo was categorized into the following intervals: 0–15, 15–30, 30–60, 60–120, 120–240, and 240–360 minutes. (TIF) [file pone.0318509.s009.tif]

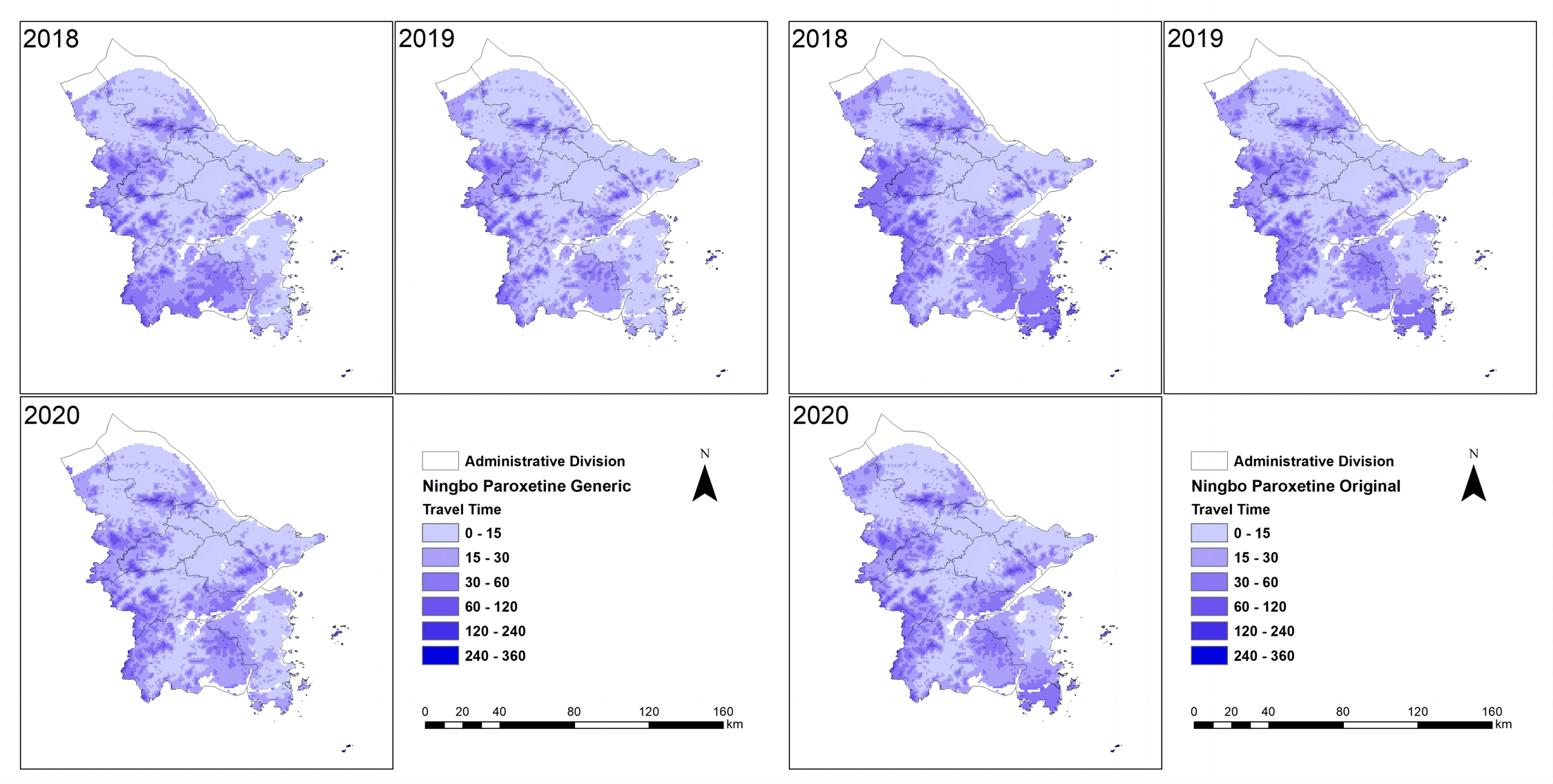

Supplement: S6 Fig — The shortest travel time from each 1 km2 population point to the nearest mental healthcare of Paroxetine in Ningbo was categorized into the following intervals: 0–15, 15–30, 30–60, 60–120, 120–240, and 240–360 minutes. (TIF) [file pone.0318509.s010.tif]

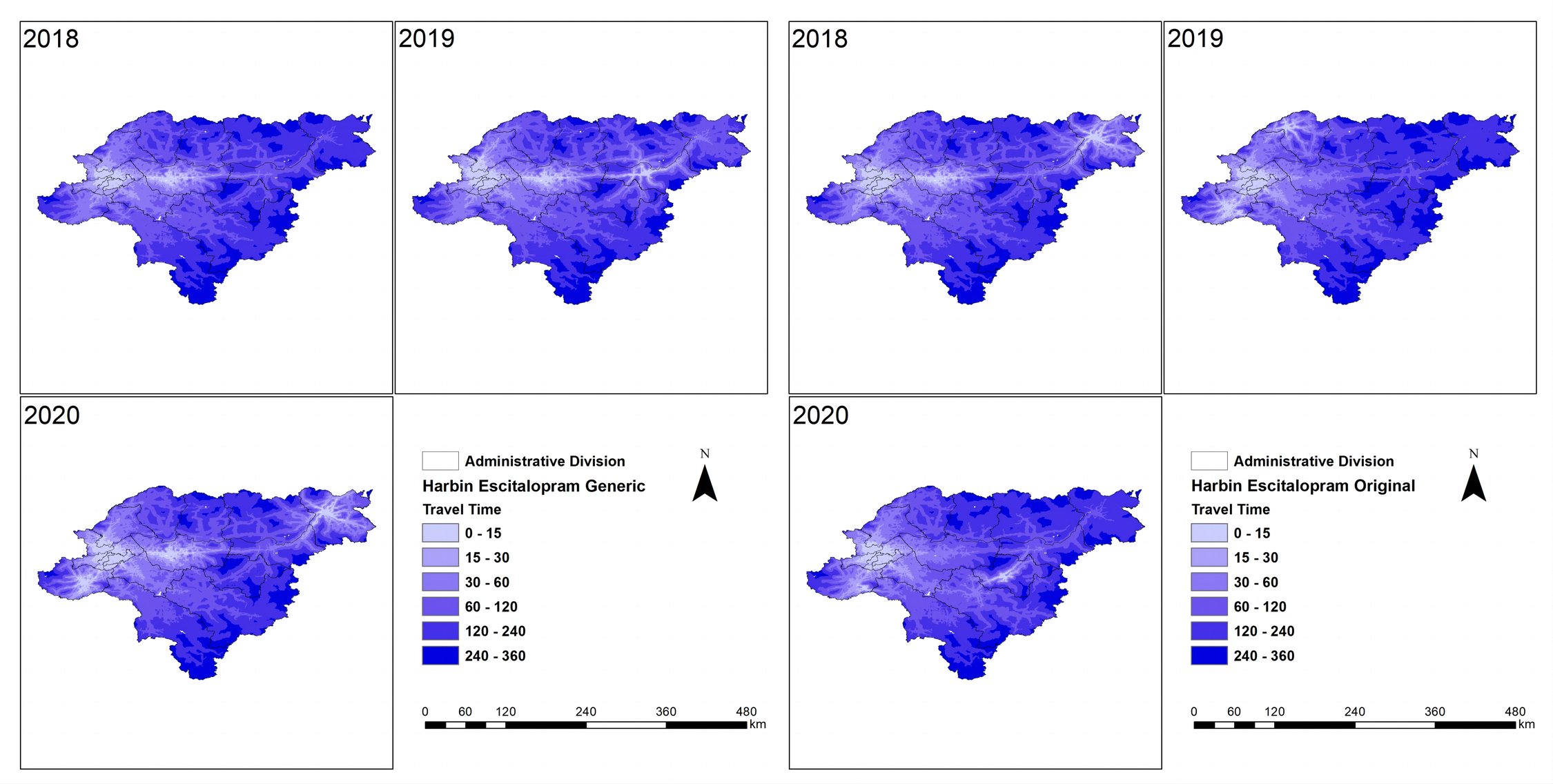

Supplement: S7 Fig — The shortest travel time from each 1 km2 population point to the nearest mental healthcare of Escitalopram in Harbin was categorized into the following intervals: 0–15, 15–30, 30–60, 60–120, 120–240, and 240–360 minutes. (TIF) [file pone.0318509.s011.tif]

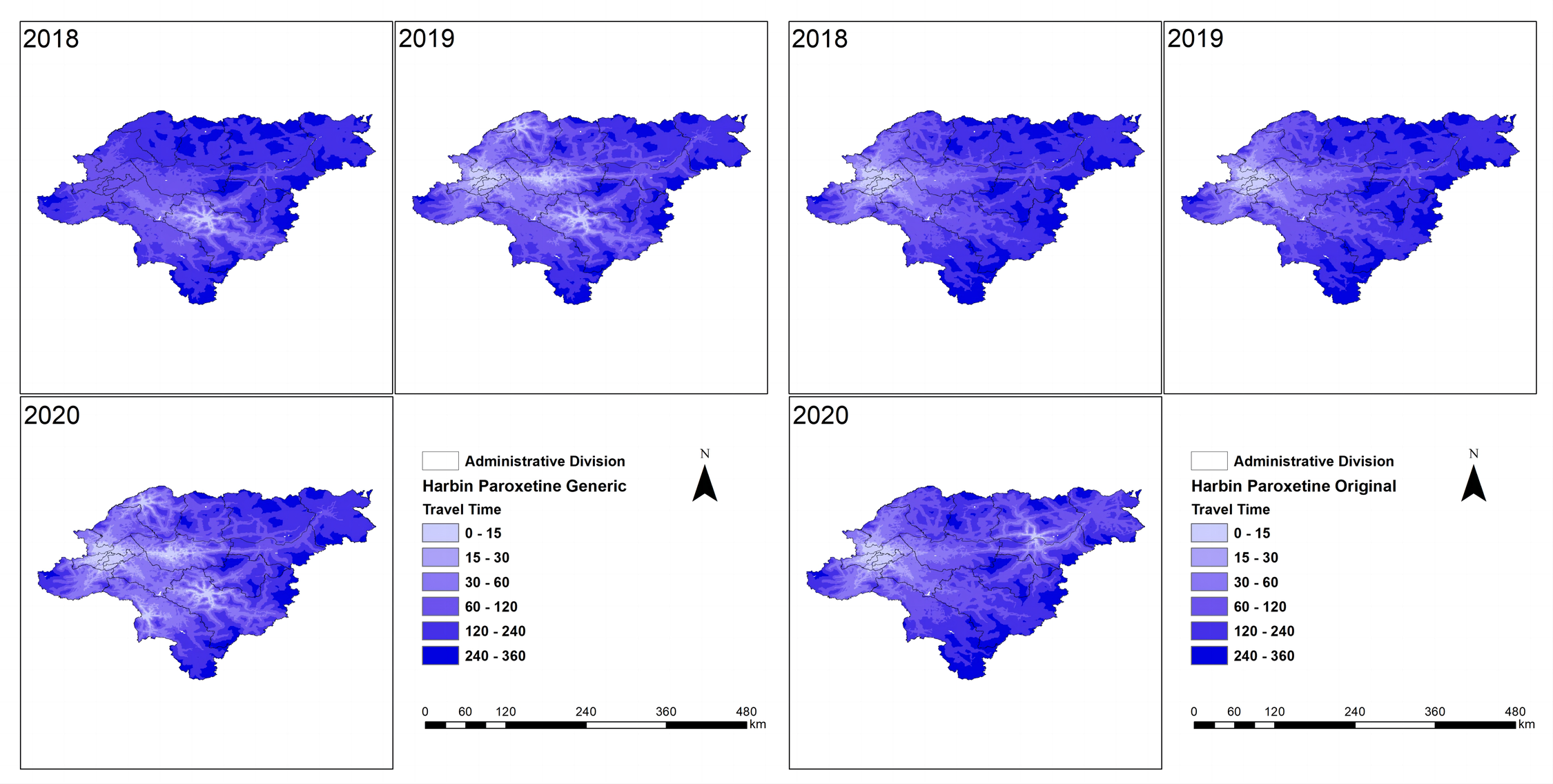

Supplement: S8 Fig — The shortest travel time from each 1 km2 population point to the nearest mental healthcare of Paroxetine in Harbin was categorized into the following intervals: 0–15, 15–30, 30–60, 60–120, 120–240, and 240–360 minutes. (TIF) [file pone.0318509.s012.tif]
