# Supplementary material for: Quantifying the impacts of volume-based procurement policy on spatial accessibility of antidepressants via generic substitution: A four-city cohort study using drug sales data
Source: PLoS One. 2025 Feb 10;20(2):e0318509. doi: 10.1371/journal.pone.0318509 (PMC11809876; doi:10.1371/journal.pone.0318509)
Supplement: Attachment — Submitted filename: Response to Reviewers.docx [file pone.0318509.s013.docx]

**Response letter**

Dear Editor of *PLOS ONE*,

We appreciate your suggestions and reviewers’ kind comments on our submission entitled " Quantifying the impacts of volume-based procurement policy on spatial accessibility of antidepressants via generic substitution: A four-city cohort study using drug sales data (PONE-D-24-32343R1). This study is the first in China to conduct spatial accessibility research using antidepressant procurement data and has yielded some meaningful results. These results may provide some insights for both domestic and international readers. Once again, I sincerely appreciate the editor's patience in reviewing and handling my manuscript. We have carefully addressed these comments and please find below the point-to-point responses.

All my best,

Jifang Zhou, MD, PhD, MPH

Associate Professor

China Pharmaceutical University

**Review Comments to the Author**

**Reviewer #1:** Thank you for the opportunity to review this well-written, interesting manuscript titled "Quantifying the impacts of volume-based procurement policy on spatial accessibility of antidepressants via generic substitution: A four-city cohort study using drug sales data". The manuscript is indeed well-written and the methodological choices are well justified. I would be happy to recommend publishing the manuscript.

**Response: Thank you very much for taking the time to review my manuscript. I am truly honored to receive your recognition. The VBP policy in China has directly impacted drug prices, procurement volumes, and other factors, but spatial accessibility is a novel perspective. Previous studies have primarily focused on the spatial accessibility of healthcare institutions at various levels, whereas our study is the first in China to use hospital procurement data to analyze spatial accessibility specifically in the field of antidepressant medications. We have also made several meaningful findings, and I believe these results will provide valuable insights for policymakers.** **I will continue to refine the content of the manuscript to meet the journal's requirements. Once again, thank you for your valuable review.**

**Reviewer #2:** In general, the manuscript appears to be technically sound. The study employs rigorous methods, including the least-cost-path algorithm, Gini coefficient, Theil index, Ordinary Least Squares (OLS), and mediation analysis to assess spatial accessibility and inequality of antidepressants. The data support the conclusions drawn, as the study found varied improvements in medication accessibility and inequality across cities, with more pronounced effects in economically less developed cities. Overall, the manuscript meets the standards for clarity, readability, and proper use of English.

**Response: Thank you very much for thoroughly reviewing my manuscript. I am also truly honored to receive your recognition of the strengths of the manuscript.** **Depression is highly prevalent in China, and patients need a prescription to purchase antidepressants from designated hospitals. Therefore, whether depression patients can access hospitals without spatial barriers is crucial. The VBP policy directly addresses drug prices and procurement volumes, but its impact on spatial accessibility and inequality remains unknown. Our study is the first in China to use hospital-level procurement data to analyze spatial accessibility in the specific context of antidepressants. We found that the substitution of generic drugs plays a significant role in improving spatial inequality. These findings are expected to provide valuable evidence for domestic policymakers and offer reference value for international research.**

**However, the manuscript does have some shortcomings. I greatly appreciate your constructive feedback and the valuable suggestions for improvement.** **I will address each of the issues you raised and make the necessary revisions, striving to meet your expectations.**

(1) The abstract should include the most significant results obtained during the research.

**Response:** **I completely agree with your suggestion, as the abstract of my manuscript is currently quite general and does not include the most significant results.** **To make the abstract clearer, I have revised it into a structured format and revised the results and conclusion sections. The revised version is as follows (Line 65 in** **Revised Manuscript with Track Changes):**

**Results: Under the influence of the VBP policy, we observed varying degrees of growth in the procurement volumes of two antidepressants across different cities (Escitalopram: Beijing 30.3%, Shanghai 26.2%, Ningbo 37.4%, Harbin 25.7%; Paroxetine: Beijing 28.2%, Shanghai 1.2%, Ningbo 50.2%, Harbin 590.5%). The increase in the procurement volumes of antidepressants across cities was primarily driven by generic drugs (Escitalopram: Beijing 159.8%, Shanghai 75.0%, Ningbo 146.4%, Harbin 146.3%; Paroxetine: Beijing 67.3%, Shanghai 4.9%, Ningbo 58.0%, Harbin 15,758.3%). In the results on spatial inequality, we observed annual improvements across all cities, with more pronounced progress in economically underdeveloped regions (Escitalopram: Gini in Harbin decreased by 10.6%; Paroxetine: Gini in Harbin decreased by 32.6%). In Beijing, the substitution of generic escitalopram was found to be a partial mediating factor in the improvement of spatial inequality (ACME = -0.00, p-value = 0.01; ADE = -0.00, p-value = 0.02). In Harbin, the substitution of generic paroxetine was identified as a complete mediating factor for spatial inequality (ACME = -0.04, p-value = 0.01; ADE = 0.01, p-value = 0.14). Conclusions: This study found that the spatial accessibility and inequality of antidepressant medications gradually improved under the influence of the VBP policy. These improvements can be partially attributed to the substitution of generic drugs.**

**In the revised version of the abstract, the main results of the study are included and specifically presented in numerical form, with a summary provided in the conclusion section.** **Thank you once again, and I hope the revised abstract meets your approval.**

(2) Line 165: "of the first round of the, paroxetine" – please check and revise this sentence for clarity and grammatical accuracy.

**Response: Thank you very much for your careful reading. This section indeed contains basic grammatical issues, which was my oversight.** **In the study area section, I intended to convey that we selected two antidepressant medications from the first batch included in the expanded VBP catalog as the subjects of this study.** **Without altering the original meaning, I corrected the grammatical issues in the sentence. The revised version is as follows (Line 198 in** **Revised Manuscript with Track Changes): In this study, we selected two depression medicines from the first round of the VBP: paroxetine and escitalopram.**

(3) In the section "Data sources," appropriate references to websites or other sources should be provided where it is possible.

**Response:** **I fully agree with your suggestion to include references to website information in the data sources section.** **This not only enhances the transparency of the research methodology but also makes the manuscript more rigorous.** **In this study, while the procurement data for medications was obtained from the Chinese Pharmaceutical Association, all other data sources are publicly available.** **This includes hospital coordinates, administrative boundaries, population information, and friction surface data.** **I have specified the URLs for each publicly available data source in the Data sources section (Line 208 in** **Revised Manuscript with Track Changes).** **For more detailed information on data sources, readers can refer to the Supporting information in S2 Table: Datasets used in this national study in China.** **Once again, thank you for suggesting this.**

(4) Line 189: It is recommended to include the formula in the main text of the manuscript.

**Response:** **Thank you very much for your reminder. The methodology section of my manuscript indeed lacks the necessary formulas to support the explanations.** **The Gini coefficient and other inequality indices were originally used to measure economic inequality in populations, but in this study, they are applied to assess spatial accessibility inequality within regions. Therefore, it is necessary to provide a specific explanation of how the parameters in the formulas are chosen, and I fully support your suggestion.** **Following your advice, I have included the formulas for the Gini coefficient and Theil index, along with the relevant explanations, in the methodology section of the main text (Line 223 in Revised Manuscript with Track Changes). I have also cited the appropriate references. This helps ensure that the calculation methods are clearly understood while maintaining the rigor of the writing.**

(5) The principles of the "volume-based procurement" strategy are not described but are valuable for a broad audience of readers.

**Response:** **Thank you very much for your suggestion from an objective perspective. I fully agree with adding a description of the principles of the VBP policy to help international readers gain a deeper understanding of the policy background.** **The VBP policy was piloted in China in 2018, following the principle of "price reduction through volume-based purchasing," and has yielded very positive results.** **To facilitate better understanding for international readers, I have used more vivid wording. The revised version is as follows (Line 132 in** **Revised Manuscript with Track Changes): The VBP policy follows the principle of "reducing prices through bulk purchasing," offering hospitals and patients more cost-effective medications.**

(6) Line 359: Should the abbreviation VBP here and afterward stand for "Value-Based Pricing" instead of "volume-based procurement"? Ensure consistency in the meanings of terms throughout the article.

**Response:** **Thank you very much for pointing out the inconsistency in the use of the VBP abbreviation. I completely agree with your perspective.** **"Volume-based procurement" is the correct expansion of VBP, and I have revised it accordingly (Line 411 in** **Revised Manuscript with Track Changes).** **"Volume-based procurement" better captures the essence of the VBP policy, as it reflects the concept of centralized bulk purchasing of medications. In contrast, "Value-Based Pricing" appears to refer more to the re-pricing of drugs.** **Similarly, I have also revised other instances of inconsistent usage of VBP throughout the manuscript to ensure such issues do not occur (Line 66, 93, 130, 198 in** **Revised Manuscript with Track Changes).** **Additionally, I ensured that the expanded form was used only when VBP first appeared in the abstract and main text, with subsequent mentions using the abbreviation. This aligns with the conventions of academic writing. Once again, I sincerely thank you for pointing out this issue in the manuscript.**

(7) Describe the applicability of the proposed methodology for conducting similar analyses in countries other than China.

**Response:** **I fully agree with your suggestion that a valuable study should provide reference value for other researchers in the field. As you mentioned, the research methodology used in this study is also applicable to similar research in other countries. Currently, studies on healthcare spatial accessibility in various countries focus on accessibility at the level of healthcare institutions, whereas research on the spatial accessibility of specific medications has not yet been developed. International scholars can refer to the methodology in this study for similar calculations of medication spatial accessibility, inequality, and related mediation analysis.**

**Therefore, I have added a section titled "Applicability in Other Countries" in the "Strength of the Present Study" part of the manuscript for other researchers to reference.** **The added content is as follows (Line 451 in** **Revised Manuscript with Track Changes):** **Similarly, this research methodology can also be extended to countries other than China. First, it is essential to determine whether the travel destination in the model is the sole means for patients to obtain the medication. To elaborate, antidepressants in China are classified as restricted medications, requiring patients to visit hospitals for a prescription. In other countries, however, antidepressants may not be regulated in the same way. In such cases, additional data on the availability of these medications in pharmacies would be required, and the same principle applies to other medications.** **Then, in conjunction with the pharmaceutical policies of the country, a mediation analysis method similar to that used in this study could be employed to explore whether factors such as "generic drug substitution" and "market concentration improvement" indirectly influence the spatial inequality of the medication.**

(8) In the Conclusion section, list the main results obtained in this study.

**Response:** **Thank you very much for repeatedly pointing out the issue of unclear main results in my manuscript. I will revise the manuscript to address these issues. Similar to the changes made in the abstract, I have also incorporated main results in the conclusion section to support my findings. The main results of this study include three aspects: first, under the influence of the VBP policy, the procurement volume of antidepressants increased, and prices decreased. Second, the spatial accessibility and inequality of antidepressants improved annually. Finally, generic drugs were proven to be an effective mediating factor for this improvement.**

**The added content is as follows (Line 488 in** **Revised Manuscript with Track Changes):** **Under the influence of the VBP policy, we observed varying degrees of growth in the procurement volumes of two antidepressants across different cities. Regarding spatial inequality, improvements were observed annually across all cities, with more significant progress in economically underdeveloped regions. In Beijing, the substitution of generic escitalopram was found to be a partial mediator in the improvement of spatial inequality. In Harbin, the substitution of generic paroxetine was identified as a complete mediator for spatial inequality.**

(9) What are the main results expressed in numerical terms?

**Response:** **Thank you very much for your valuable suggestion. I will make every effort to revise my manuscript accordingly. I understand that this feedback is similar to your earlier comments about the lack of clarity regarding the main research results in the abstract and conclusion. As a result, I have added quantified main results in both sections, with more detailed findings presented in the results section and tables (Line 76, 440 in** **Revised Manuscript with Track Changes). However, I only added numerical results in the abstract section, while in the conclusion section, I summarized the main findings of the article in a more concise manner. I hope these additions will help clarify the key findings of the study and provide readers with a clearer understanding of the research.**

**Your feedback has been extremely helpful, and I truly appreciate your valuable comments. Thank you once again for your thoughtful review.**

**Journal requirements:**

(1) Please ensure that your manuscript meets PLOS ONE's style requirements, including those for file naming.

**Response:** **Thank you for the editorial comments and suggestions. I will carefully revise the manuscript to meet the journal's requirements.** **I have made the necessary revisions to the writing style based on the *PLOS ONE* guidelines, including adjustments to the font, font size, paragraph indentation, and other formatting requirements. I have also updated the file type and name as per the journal's specifications. If I have overlooked any style issues, I would greatly appreciate your guidance and feedback. Thank you very much.**

(2) Please note that PLOS ONE has specific guidelines on code sharing for submissions in which author-generated code underpins the findings in the manuscript. In these cases, we expect all author-generated code to be made available without restrictions upon publication of the work.

**Response:** **Thank you for pointing out the issue. I fully respect the guidelines on code sharing for submissions.** **The authors declare that the code used in this study will be made available without restriction upon reasonable request.** **Once again, thank you for your understanding and helpful reminders.**

(3) We note that the grant information you provided in the ‘Funding Information’ and ‘Financial Disclosure’ sections do not match. When you resubmit, please ensure that you provide the correct grant numbers for the awards you received for your study in the ‘Funding Information’ section.

**Response:** **Thank you very much for your reminder. After reviewing, we found that the statement 'The author(s) received no specific funding for this work' submitted during the application is incorrect, as this study was supported by relevant funding. However, I have not found an option in the current system to correct this statement. Could you please assist in modifying it in the backend? Thank you very much. Additionally, upon further checking, there are some minor issues with the fund numbers. I have already updated both in the system and in the manuscript (Line 43 in** **Revised Manuscript with Track Changes), and the correct funding information is now as follows: This work was supported by the National Natural Science Foundation of China (72064032 and 72274008); Natural Science Foundation of Beijing Municipality (9232010); Natural Science Foundation of Inner Mongolia Autonomous Region, China (2024MS07007).**

(4) Please provide a complete Data Availability Statement in the submission form, ensuring you include all necessary access information or a reason for why you are unable to make your data freely accessible. If your research concerns only data provided within your submission, please write "All data are in the manuscript and/or supporting information files" as your Data Availability Statement.

**Response:** **Thank you very much for your suggestion. I fully agree with your view on modifying my data availability statement. In this study, the ownership of the drug procurement data belongs to the China Pharmaceutical Association, while all other data are from publicly available resources. Based on your suggestion, I have added the means to access the original data in the statement (Line 52 in** **Revised Manuscript with Track Changes):** **Data cannot be shared publicly because they are owned by the Chinese Pharmaceutical Association. Data are available from the Chinese Pharmaceutical Association Science and Technology Development Center (contact via Xu Yingfeng, +8610-65662756) for researchers who meet the criteria for access to confidential data.**

(5) PLOS requires an ORCID ID for the corresponding author in Editorial Manager on papers submitted after December 6th, 2016. Please ensure that you have an ORCID ID and that it is validated in Editorial Manager. To do this, go to ‘Update my Information’ (in the upper left-hand corner of the main menu), and click on the Fetch/Validate link next to the ORCID field. This will take you to the ORCID site and allow you to create a new ID or authenticate a pre-existing ID in Editorial Manager.

**Response:** **Thank you very much for your reminder. I fully understand the requirement to link the corresponding author's ORCID for submission.** **Due to our corresponding author previously linking their ORCID to a reviewer account for PLOS ONE, the submission account could not be linked to the ORCID again. I have resolved this issue by unlinking ORCID from the previous reviewer account and linking it to the current submission account. Thank you very much for your reminder and patience. Please check if everything now meets the requirements.**

(6) We note that Figures 1, 2 ,3, 4, S1, S2, S3, S4, S5, S6, S7 and S8 in your submission contain [map/satellite] images which may be copyrighted. All PLOS content is published under the Creative Commons Attribution License (CC BY 4.0), which means that the manuscript, images, and Supporting Information files will be freely available online, and any third party is permitted to access, download, copy, distribute, and use these materials in any way, even commercially, with proper attribution. For these reasons, we cannot publish previously copyrighted maps or satellite images created using proprietary data, such as Google software (Google Maps, Street View, and Earth).

**Response:** **Thank you very much for your reminder. The source and copyright issues of the map information do indeed require careful attention.** **However, there are no copyright issues with any of the figures in the current manuscript.** **Firstly, the administrative boundaries in the maps we created are sourced from the Chinese Administration of Surveying Mapping and Geoinformation, with the review number “GS (2022) 3995号”. In geographical studies in China, all maps used for publication are based on administrative boundaries provided by the Chinese Administration of Surveying Mapping and Geoinformation. Therefore, this source is public and does not involve any copyright issues. Secondly, the other population and transportation data in the maps are also from publicly available resources, as detailed in S2 Table.** **Following your reminder, I have added a Copyright notice on the title page, specifying the source of the maps and the review number (Line 57 in** **Revised Manuscript with Track Changes).**

(7) Please include captions for your Supporting Information files at the end of your manuscript, and update any in-text citations to match accordingly.

**Response:** **Thank you very much for your suggestion. This part was indeed an issue with the formatting of my submitted manuscript. It was lacking a statement regarding supporting information provided.** **I have followed the PLOS ONE writing guidelines and added a "Supporting Information" section at the end of the manuscript.** **I have listed all the titles of the supplementary tables and figures, ensuring each is properly referenced and corresponds to the respective section (Line 582 in** **Revised Manuscript with Track Changes).**

(8) Please review your reference list to ensure that it is complete and correct. If you have cited papers that have been retracted, please include the rationale for doing so in the manuscript text, or remove these references and replace them with relevant current references. Any changes to the reference list should be mentioned in the rebuttal letter that accompanies your revised manuscript. If you need to cite a retracted article, indicate the article’s retracted status in the References list and also include a citation and full reference for the retraction notice.

**Response:**

**Thank you very much for pointing out the issues in the references section of my manuscript.** **I have reviewed all the references in my manuscript and found that one of the titles was indeed inaccurate.** **It is the eighth reference in my manuscript. I have corrected it to the following (Line 541 in** **Revised Manuscript with Track Changes):** **Wang X, He X, Zhang P, Zhang M, Ma R, Dai R, Li X. The impact of the national volume-based procurement policy on the use of policy-related drugs in Nanjing: an interrupted time-series analysis. Int J Equity Health. 2023 Sep 28;22(1):200.** **The other cited references in the manuscript are all currently publicly accessible and have not been retracted.** **Thank you once again for your valuable reminder.**

**I hope the above revisions improve my manuscript, and I truly appreciate your patience and understanding. I sincerely look forward to your response.**
